# Buried iceberg scours reveal reduced North Atlantic Current during the stage 12 deglacial

**DOI:** 10.1038/ncomms10927

**Published:** 2016-03-16

**Authors:** Andrew M. W. Newton, Mads Huuse, Simon H. Brocklehurst

**Affiliations:** 1 School of Earth, Atmospheric and Environmental Sciences, University of Manchester, Manchester M13 9PL, UK

## Abstract

Reconstructing past ocean-climate environments and heat transport requires proxies from which these conditions can be quantified. This is particularly important for the evaluation of numerical palaeoclimate models. Here we present new evidence for a reduced North Atlantic Current (NAC) at the termination of the third last glacial, for which palaeocurrent information was previously unavailable. This is based on an exquisitely preserved set of buried iceberg scours seen in three-dimensional seismic reflection images from the mid-Norwegian slope. The scours were formed ∼430 ka during the transition from glacial to interglacial conditions. The spiral geometry of the scours suggests that they were carved by grounded icebergs influenced by tidal and geostrophic ocean currents. Using the ratio between the estimated tidal and geostrophic current velocities and comparing them with velocities from the Last Glacial Maximum and the present, we show that the stage 12 NAC velocities may have been ∼50% slower than the present.

## Abstract (web summary)

Reconstructing past ocean conditions, particularly current velocity, is difficult due to a lack of available proxies. Here, the authors present a set of well-preserved buried iceberg scours from the mid-Norwegian slope, from which they are able to infer North Atlantic current speeds during the third-last glacial.

## Graphical abstract

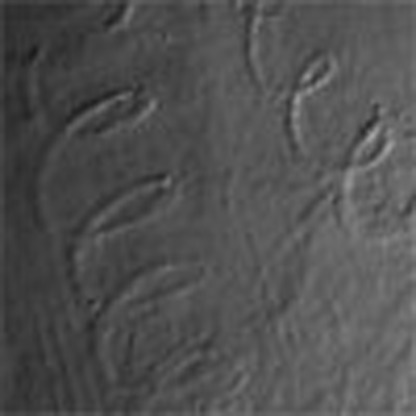

Large areas of Northwest Europe have been affected by major glaciations repeatedly throughout the Pleistocene^1^. As an area of extensive oil and gas exploration, the mid-Norwegian shelf and slope is one of the most comprehensively studied glaciated margins in the world^1^. The mid-Norwegian shelf has a series of banks cut by cross-shelf troughs and the slope represents margin progradation by trough-mouth fans and intervening shelf-slope prisms (Fig. 1). The bedrock is mainly Triassic to Cenozoic in age, while the late Cenozoic succession comprises a sedimentary wedge deposited at greatly accelerated rates during the Pleistocene glaciations^1^. The Plio-Pleistocene Naust Formation is predominantly composed of glacial deposits with occasional sandy interbeds up to a few metres thick^1^, and consists of five tentatively dated sediment packages^2^,^3^. Dipping topset strata indicate post-depositional subsidence of the outer and middle shelf^1^ (Fig. 2).

Although most published work has concentrated on the last glacial maximum (LGM), the offshore record in the Norwegian Sea shows repeated glaciations for at least the entire Pleistocene^4^,^5^,^6^. Understanding the environmental changes through different glacial–interglacial cycles allows for a better understanding of ice-age chronology and extent. This is crucial for the validation of numerical palaeoclimate models^7^ and our understanding of historic, present and future sea level variability, ocean dynamics, ice sheet stability and climate change^8^,^9^,^10^,^11^,^12^,^13^,^14^. Ocean dynamics, for which very little data exist, are a key constituent of these models. Traditional proxies for ocean-current velocities and water mass origins include grain-size measurements and provenance studies^15^,^16^. Palaeocurrent directions have also been inferred from furrows, ice-rafted detritus and iceberg scours^17^.

The North Atlantic Current (NAC) is a warm ocean current that is part of the Atlantic Meridional Overturning Circulation (AMOC)^14^,^18^ and has North Atlantic Deep Water production (NADW) at its northern extremity. In the Norwegian Sea, the NAC splits into the Norwegian Atlantic Slope Current (NASC) and the Norwegian Atlantic Front Current (NAFC) (Fig. 1). NADW is produced at the high latitudes of the Nordic Seas before returning south at depth over the Greenland-Scotland ridge^19^. The NASC is a wind-driven current^20^ steered by the topography of the slope that flows through our study area. The present-day mean velocity of the NASC current^21^ is 20–40 cm s^−1^ while tidal currents of 20–30 cm s^−1^ are also recorded here^22^.

The AMOC has been an important area of research because of its fundamental role in the transport of heat from the tropics to high latitudes^23^, and thus its influence on global climate^14^,^24^,^25^. Little is known about ocean currents during past glacials or interglacials, but it has been suggested that three modes of NADW formation may exist; the modern, the glacial and the Heinrich mode^19^,^26^,^27^,^28^. Some numerical experiments have suggested a small amount of NADW being produced in the Nordic Seas by brine rejection and buoyancy loss during the glacial mode^29^. However, the majority of numerical modelling studies of the glacial mode have shown the same general trends for a reduced NADW^30^,^31^,^32^,^33^ that formed further south^34^,^35^,^36^,^37^,^38^,^39^ and with a much reduced northward advection of heat.

The Heinrich mode is associated with the freshening of North Atlantic surface waters by significant inputs of icebergs and meltwater^38^,^39^,^40^,^41^,^42^. This is suggested to have previously caused the collapse of the AMOC^25^ and subsequently allowed Antarctic-derived bottom waters to reach depths as shallow as 1,000 m in the North Atlantic^36^,^39^. Despite general agreement, these models still show considerable variation on the exact setup of the thermohaline current and rates of overturning^35^,^43^,^44^,^45^, which often disagree with the limited, North Atlantic biased and often complicated proxy records^19^,^28^. The NAC is thus an important component of past, contemporary and future dynamics of the thermohaline circulation and is the focus of this study.

In this paper, the glacial geomorphology of a palaeo-seafloor dated to ∼430 ka is used to reconstruct ocean dynamics in the Norwegian-Greenland Sea during the stage 12 deglacial. Spiral-shaped iceberg scours show that the ratio between the tidal and geostrophic currents is markedly different from the present and indicate that the northward advection of heat through the NAC may have been limited by a halving of current velocities relative to the present.

## Results

### Seismic geomorphology

**Figure 1:**
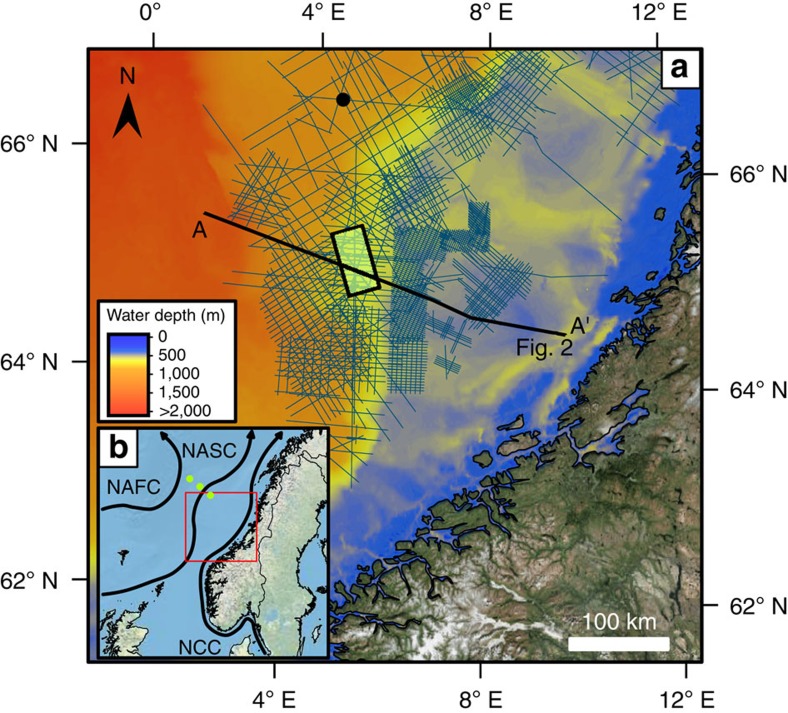
Study site area. (**a**) Distribution of two-dimensional seismic lines (blue lines) and 3D volume (green box with black outline) over GEBCO bathymetry for the mid-Norwegian shelf. Satellite imagery of the hinterland is from the ‘World Imagery' layer available from ArcMap online. Location with respect to the Norwegian mainland is shown in **b** in the red box. Black dot indicates ODP site 644. Location of seismic line in Fig. 2 is indicated by a thick black line. (**b**) Major surface currents offshore Northwest Europe^21^. NAFC, Norwegian Atlantic Front Current; NASC, Norwegian Atlantic Slope Current; NCC, Norwegian Coastal Current. Three green dots show locations of ODP sites 642–644 used for ice-rafted detritus analysis^4^.

Investigations using three-dimensional (3D) and two-dimensional seismic reflection data from the mid-Norwegian slope (Fig. 1) were conducted to constrain oceanographic conditions during the Marine Isotope Stage (MIS) 12 to MIS 11 transition. Present-day seafloor depths across the 3D survey are 550–940 m, with a mean depth of 720 m. Seismic reflections representing palaeo-seafloor surfaces were extracted for glacial geomorphological analysis. A surface with landforms interpreted as iceberg scour marks at the top of the Naust U package was dated to ∼430 ka based on a spike in ice-rafted detritus^4^ at three ODP sites on the Vøring Plateau (Fig. 1). This date marks the transition from glacial conditions during MIS 12 to interglacial conditions in MIS 11. This transition is important because the MIS 11 interglacial is viewed as a good proxy for the current interglacial^46^ and how ocean currents respond to large fluxes of glacial meltwater.

Subsidence rate estimates^47^,^48^ in the area are 1.2 m ka^−1^ for the last ∼300 ka. This rate is based on the presence of buried iceberg scours and ice sheet grounding lines, and comparison with contemporary examples on the mid-Norwegian shelf seafloor to suggest maximum water depths (∼500 m) for these features^47^,^48^. Although glacial landforms have been found in greater water depths than in the present, they are rare. Assuming this subsidence rate occurred throughout the time period since formation of the scours, the palaeo-seafloor depth can be estimated. At ∼430 ka, the global sea level was ∼115 m lower than the present^10^. Using this sea level stand and the subsidence rates inferred from glacial landforms^47^,^48^ gives an estimated palaeo-seafloor depth in the area of ∼250–300 m. However, geometrical analysis of the palaeo-shelves and the assumption that they were deposited near-horizontally provides a larger estimate of palaeo-water depths of ∼400-500 m for the scoured surface. Although this is not of major importance for the implications of this study, this highlights the uncertainty in the use of glacial landforms to infer water depths and post-depositional subsidence.

**Figure 3:**
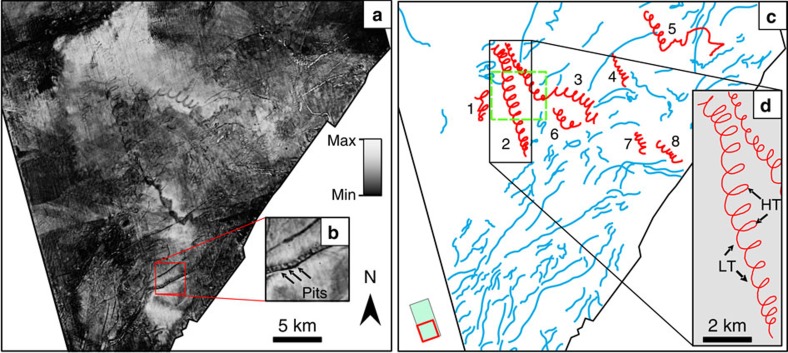
Seismic geomorphology. (**a**) Surface peak amplitude map derived from the 3D volume, which highlights the iceberg scours from the MIS 12 deglaciation. Grey scale bar represents the peak seismic amplitude extracted across the 3D surface. For geographic location, see Fig. 1; and for stratigraphic location, see Fig. 2. Enlarged images of the amplitude and depth changes across the surface are provided as Supplementary Figs 1 and 2. (**b**) Enlarged image of pits formed by the crater chain iceberg. (**c**) Digitized scour geometry from **a**. Red digitized scours are those demonstrating variable tidal and geostrophic current effects. Blue lines show linear and curvilinear iceberg scours. Numbers locate scours referred to in the text and Supplementary Table 1. Green dashed box shows the location of Fig. 4. (**d**) Scour 2 trajectory used for spring tide analysis in Supplementary Fig. 3. HT and LT represent the high and low tides, respectively that were used for estimating tidal and geostrophic current velocities.

**Figure 4:**
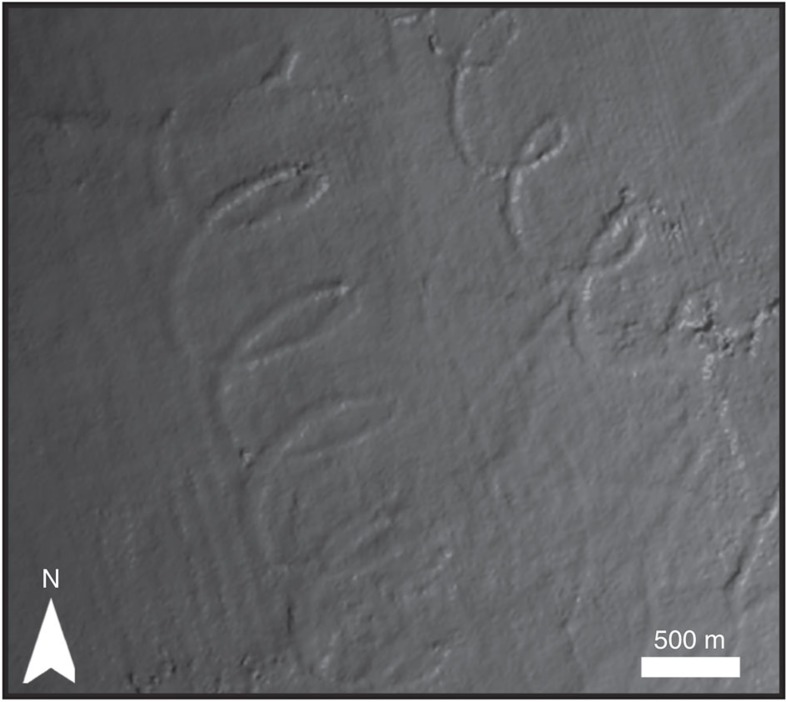
Spiral geometry. Shaded relief image showing the spiral geometry of the iceberg scours. For location, see green box in Fig. 3c. Surface is vertically exaggerated by 15 to emphasize the geometry of these subtle features. Acquisition footprint is also visible and orientated north-northeast to south-southeast. Grey scale values between 1,360 and 1,420 ms TWT.

**Figure 5:**
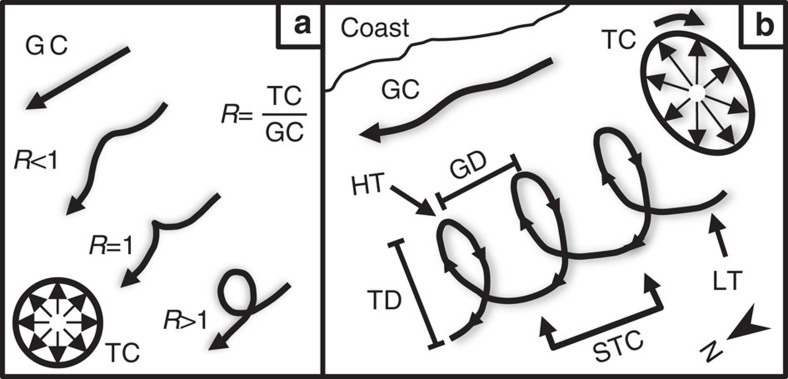
Iceberg motion with currents. (**a**) Conceptual model of the interaction between the tidal rose and geostrophic currents and how the ratio influences iceberg trajectory. Figure adapted from ref. 50. GC and TC are the geostrophic and tidal currents, respectively. (**b**) Synthesis model showing the key variables used to calculate tidal and geostrophic current velocities from the iceberg scours. A hypothetical tidal ellipse (TC) is displayed with the major axis orthogonal to the geostrophic current. TD is the Tidal Distance (referred to as amplitude in the text) covered by the iceberg and is used to calculate tidal velocities based on a semi-diurnal tidal period. GD is the Geostrophic Distance (referred to as wavelength in the text) used to calculate geostrophic current velocities. HT and LT represent the locations on the tidal cycle for high and low tide, respectively. STC is the semi-diurnal tidal cycle of 12 h 25 min that is used to estimate iceberg velocities.

The palaeo-seafloor surfaces are dominated by linear and curvilinear scours oriented southwest–northeast (Fig. 3). Of particular interest here is a set of spectacular spiral-shaped iceberg scours (Fig. 4). There are eight well-developed tracks from 5.5 to 31.5 km long (Supplementary Table 1), whose geometry varies between large loops and sharp, arcuate changes in direction. Scour widths vary more for the linear and curvilinear scours (76–268 m range with a mean width of 132 m) than for the spiral scours (88–120 m with a mean width of 104 m). Of the eight spiral scours studied, six have positive and two negative reliefs on the mapped surface. Positive relief is due to some scours being filled with turbidite sands, which cause differential compaction ridges relative to surrounding muddy sediments and imaging limitations imposed by the vertical seismic resolution (∼10 m). The spiral scours are interpreted as having been carved by icebergs driven by tidal and geostrophic currents, similar to free-floating iceberg drift tracks recorded off Antarctica^49^ and eastern Canada^50^. The icebergs were moved by the net effect of the two currents impacting upon them. A simple model of the dynamics shows that the ratio between the geostrophic and the tidal current velocities can be used to determine the geometry of the iceberg trajectories (Fig. 5a). When the ratio is greater than unity, the icebergs tend to move in spiral drift tracks, as shown by scour 2 (Fig. 3). If the ratio moves closer to unity or less, the loops are stretched to more arcuate trajectories marked by sharp changes in direction. However, as described below, certain conditions must be present for the spiral trajectory to occur.

### Iceberg and current dynamics

Although similar geometries have been shown for the drift patterns of icebergs in the Labrador Sea and Antarctica^49^,^51^, they have not been analysed in the context of the oceanographic dynamics that are required to form such trajectories. In the Northern Hemisphere, the Coriolis effect deflects moving objects to the right of their travel path. This process works together with tidal advection such that the current can move in complex tidal ellipses that can be either clockwise or counter-clockwise and can vary in shape from circular to rectilinear^52^.

Contemporary tidal ellipses in the study area are clockwise with the major axis generally oriented west to east^53^. No data are available for tidal ellipses in the area during any previous glaciation. In this instance, to form the spiral iceberg trajectory requires the major axis of the tidal ellipse to be orthogonal to the geostrophic current (Fig. 5b) and for the tidal current to be the more dominant. This would allow the elliptical trajectory of the iceberg through a tidal cycle to be stretched by the geostrophic current to form the spirals. Presently the tidal ellipse^53^ in the area is similar to that which is hypothesized for the formation of these spiral scours. The only major difference between the present-day and the ∼430 ka currents estimated from the spiral-shaped scours is that the geostrophic current dominates over the tidal currents in the present^20^, whereas during the MIS 12 deglacial the opposite appears to have been true.

An important issue arises as to why the spiral features show a tidal signal, but the scours oriented southwest–northeast do not (Fig. 3). This difference may be attributed to variability in iceberg geometries and masses, and the tidal ellipse geometry. The linear and curvilinear iceberg scours may have been formed by icebergs so large that their inertia meant they did not respond to changes in current velocity and direction on a tidal timescale. The different scour geometries may also be the result of a different ratio between the current velocities (Fig. 5) at a different time during the glacial–interglacial cycle. Thus, although the scours are observed on the same surface, they may have been formed by different current regimes during this transition. Minor wiggles in the curvilinear and linear scours suggest a tidal influence, but determining relative ages through cross-cutting relationships is not possible due to limitations in seismic resolution.

The tidal ellipse geometry is also crucial for developing the spiral-shaped movements of the icebergs. Tidal ellipses can vary significantly in their geometry from circular to rectilinear^52^, so if the two currents were not orthogonal to each other as described above then the resulting geometry of the trajectory would likely be more chaotic. Even if the major axis of a rectilinear ellipse was orthogonal to the geostrophic current, the sharper changes in the tidal vectors would result in an iceberg trajectory less smooth than the spirals presented here. This may explain the variability of the scour trajectories and more broadly the rarity of observations of spiral scours. Their formation requires the tidal velocities to be greater than the geostrophic current, particular iceberg geometries and amenable water depths. It would also require that the tidal ellipse has an elliptical geometry that is correctly orientated with respect to the geostrophic current for the tidal signal to produce spiral-shaped seabed scours.

Given that each loop in the spiral represents a tidal cycle, and there are approximately two tidal cycles per day, the duration of iceberg grounding can be calculated. This varies from just 1 day (scour 1) to over a week (scour 3). One further piece of evidence for these icebergs being tidally influenced comes from the change in amplitude (Fig. 5) shown by scour 2 (Fig. 3d), which increases to a central high in the middle of the scour trajectory before reducing again (Supplementary Fig. 3). We interpret this as progression towards and then following on from the spring tide.

One of the iceberg scours shows a series of connected pits (Fig. 3b). Two possible mechanisms are proposed for its formation. The first is that the feature is a crater-chain scour formed by a partially grounded iceberg being driven landward by ocean currents and oscillating vertically in response to ocean swell^54^ from the Atlantic Ocean in the southwest. These icebergs were probably calved by the Norwegian Channel Ice Stream in the south and grounded as the main geostrophic current moved them northward up the continental slope into slightly shallower waters. The second involves the lift-off and dump of the iceberg through the tidal cycle during flood and neap conditions. Each mechanism is plausible but the crater-chain interpretation is preferred due to its morphological similarity to crater chains described elsewhere^54^. If the pits were the result of tidal activity then it is possible that between the tidal floods when the iceberg is lifted from the floor the iceberg might move at different rates. Subsequently, when the iceberg is grounded again during the ebb tide the distance between the pits would not be as regular as is shown in this example.

### Reconstructing ocean currents

The trajectories of large, deep-draft icebergs are predominantly determined by ocean currents, rather than the wind, and can thus be used to understand palaeocurrents in our study area. Using the iceberg scours and assuming a semi-diurnal tidal cycle (period of 12 h 25 min) as a time constraint, the ratio between surface tidal and geostrophic current velocities in the Norwegian Sea during the MIS 12 deglaciation was estimated (see ‘Methods' section). The landward peak of the scour wavelength (see Fig. 5) is interpreted as the high tide and the seaward trough the low tide (Fig. 3d). Scours 2 and 3 have a combined mean wavelength of 660±37 m yielding a mean geostrophic current velocity of 1.5±0.1 cm s^−1^. The combined mean amplitude for the two drift tracks is 676±37 m, which corresponds to a mean tidal current velocity of 3.0±0.2 cm s^−1^. Maximum and minimum velocity estimates are shown in Supplementary Table 1. If all of the time series are included to estimate tidal and geostrophic velocities, the values show a mean geostrophic velocity of 1.3±0.1 cm s^−1^ and tidal current velocity of 2.7±0.1 cm s^−1^.

## Discussion

The ratio between the estimates of the contemporary tidal^22^ and geostrophic^21^ current velocities is close to unity with the peak geostrophic current velocities up to 10 cm s^−1^ higher^20^. The velocities calculated from the MIS 12 spiral iceberg scours show a ratio of 2:1, with tidal currents dominating. Necessitated by the absence of modelled tidal velocities in our area for the MIS 12 deglacial, we use modelled velocities^55^ from the Norwegian Channel area for the early stages of deglaciation after the LGM from ∼300 km south of our study area. Although not a perfect analogue, the contemporary tidal velocities in our study area are very similar to those of the southern area, supporting the use of these early LGM deglacial tidal velocities as analogues for the MIS12 deglacial in the absence of more proximal data. The modelled LGM tidal velocities^55^ are similar to contemporary velocities^22^ in the area, implying that the MIS 12 ratio of 2:1 can be attributed to a geostrophic current velocity reduction of ∼50% relative to the present rather than to an increase in tidal velocities.

The icebergs that carved these scours formed when Northwest Europe was deglaciating ∼430 ka. Numerical modelling studies have shown that full glacial conditions can shift deep water formation to lower latitudes^37^, so that less oceanic heat is transferred to the Arctic. The NAC is therefore an important component for determining the relative strength and location of the NADW. The contemporary current in the area (the NASC) is steered by the topography of the shelf slope. The orientation of the geostrophic current preserved in the spiral scours trajectory shows that it was following a similar trajectory as it flowed parallel to the contours of the palaeoslope. Thus, the reduced geostrophic current likely represents a reduced NASC and more broadly a reduction in heat advection to the North Atlantic through the NAC in response to NADW formation at lower latitudes. Whether the reduction in the NAC is one event associated with the Heinrich mode of circulation^19^ or an example of the glacial mode^19^ in the North Atlantic is not clear. The Heinrich mode is, however, supported by the large number of deep-draft (∼250–500 m) icebergs in the area. Evidence for high levels of carbonate dissolution indicates cooler conditions with inhibited deep water formation in the Norwegian–Greenland Sea at a similar time^56^. The potential freshwater and iceberg input from a deglaciating European and/or Greenland ice sheet^57^,^58^ may have been enough to set off this chain of events.

Determining whether the signal from the iceberg scours was a persistent feature during glacial times with reduced NAC velocities (glacial mode of circulation); a temporary perturbation caused by an ice shelf collapse and reduction of northward heat transport by the NAC (Heinrich mode of circulation); or a progression toward the modern mode of circulation during the glacial–interglacial transition and speed up of a reduced NAC, requires more complex modelling of these features and their trajectories under different ocean-climate conditions. Nevertheless, the first-order observations of this study are robust and indicate a markedly different current regime ∼430 ka. More examples of well-preserved scours, such as those presented here, can be used to better determine palaeocurrent regimes and also the finer intricacies of the oceanographic signals that their trajectories preserve. Buried iceberg scours could thus provide an important palaeoclimatic resource for evaluating hindcast climate models. They could also help to inform us better on glaciological reconstructions and palaeoceanography, particularly when there are limited proxies available for these types of ocean dynamics. Although care needs to be taken to infer a major oceanographic event from one study site, these data provide a new and interesting insight into ocean-climate evolution for a poorly understood but crucial time period for understanding past climatic change. Importantly, 3D seismic data are becoming ever more widely accessible for glaciated areas, and future studies will allow for greater perspectives on the past, present and future climate characteristics as the number of palaeo-records of climate change increases.

## Methods

### Seismic glacial geomorphology

The seismic data were analysed using Petrel 2013.4 and Paleoscan software courtesy of Schlumberger and Eliis, respectively. The 3D and two-dimensional seismic data were converted to zero-phase before an iterative approach was used to pick a large number of seismic reflections to extract chronostratigraphic palaeo-seafloor surfaces. These horizons were used to create surfaces for a glacial geomorphological analysis. A number of different attributes were extracted across the surfaces to image features. RMS amplitude, curvature, variance and peak seismic amplitudes were investigated. Although each type of attribute imaged the spiral features, the peak seismic amplitude across the surface showed the features with the greatest clarity. This surface was then imported into ESRI ArcMap where the scours were digitized.

### Reconstructing currents from iceberg scours

The spiral-shaped iceberg scour tracks reflect a combination of both tidal and geostrophic currents. The landward peak on the scour is interpreted as the high tide, and the seaward trough as the low tide (Fig. 5). This is because as the tidal cycle progresses to the high tide, the tidal current direction is towards the coast. The change in tidal current direction is then reflected at high tide as the iceberg movement changes direction. The low tide is marked by the tidal currents moving water masses and the iceberg away from the coast. To separate these components, the straight-line distance (wavelength) travelled from one high tide peak to another is used to estimate the geostrophic current. The amplitude is used to describe the estimated lateral displacement of the iceberg during one tidal cycle. The wavelength and amplitude of the spiral iceberg scours were used to estimate the tidal and geostrophic current velocities over one semi-diurnal tidal period of 12 h 25 min. Although there would clearly be added mass through the envelope of water around the draft of the iceberg, we assume that this drag would be similar for both the geostrophic and tidal currents and thus does not influence the ratio between the two.

## Additional information

**How to cite this article:** Newton, A. M. W. *et al*. Buried iceberg scours reveal reduced North Atlantic Current during the stage 12 deglacial. *Nat. Commun.* 7:10927 doi: 10.1038/ncomms10927 (2016).

## Supplementary Material

Supplementary Information Supplementary Figures 1-3 and Supplementary Table 1

## Figures and Tables

**Figure 2:**
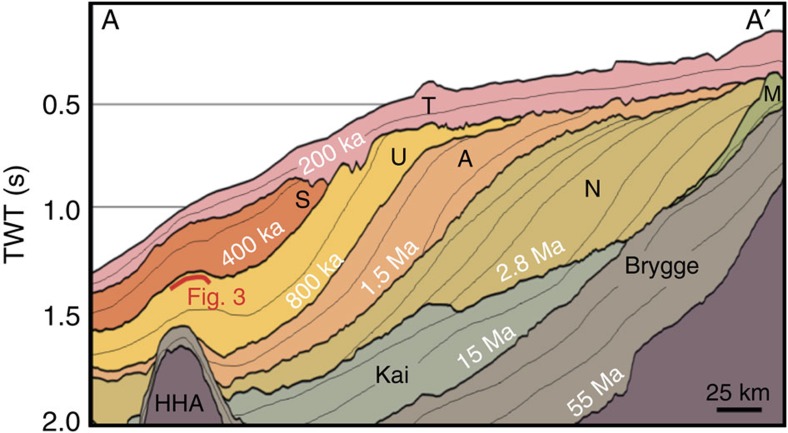
Naust Formation stratigraphy. For location, see Fig. 1. The Naust Formation is composed of Late Pliocene to Pleistocene sediments that form a thick succession of prograding sediment wedges and sheet-like units, which are mainly of glacial origin. The formation commonly down-laps onto the deltaic Kai and Molo (M) formations, as well as the Helland Hansen Arch (HHA). It is divided into five dated sequences (N, A, U, S and T) based on the limited availability of high-quality core. White writing shows the ages for the base of each package, although the dating is particularly uncertain for the older packages^3^,^59^,^60^. The red line shows the stratigraphic location of the palaeo-seafloor (Fig. 3) investigated here. The seismic profile is 70 times vertically exaggerated to illustrate large-scale margin architecture. Vertical axis is two-way travel (TWT) time. In water, 1 s TWT≈0.75 km, assuming a sound velocity of 1.5 km s^−1^; in sediments, 1 s TWT≈1 km, assuming a sound velocity of 2 km s^−1^.
