# Supplementary material for: Buried iceberg scours reveal reduced North Atlantic Current during the stage 12 deglacial
Source: Nat Commun. 2016 Mar 16;7:10927. doi: 10.1038/ncomms10927 (PMC4799367; doi:10.1038/ncomms10927)
Supplement: Supplementary Information — Supplementary Figures 1-3 and Supplementary Table 1 [file ncomms10927-s1.pdf]

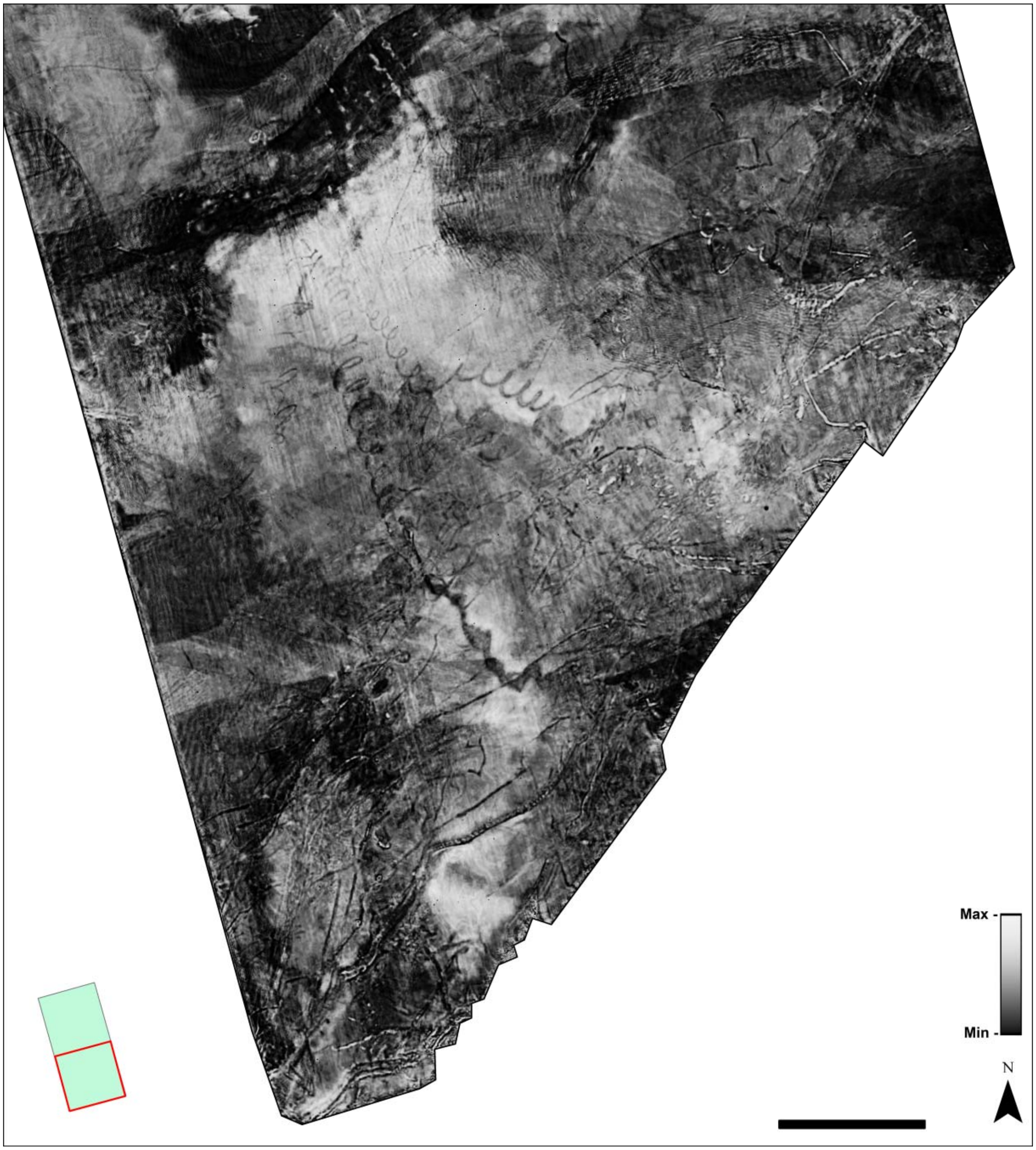

Supplementary Figure 1:

Enlarged map. Iceberg scoured horizon picked from the 3D volume showing the spiral trajectories of the icebergs. Greyscale bar represents peak seismic amplitude changes across the 3D surface. Black bar is scale bar of 5 km.

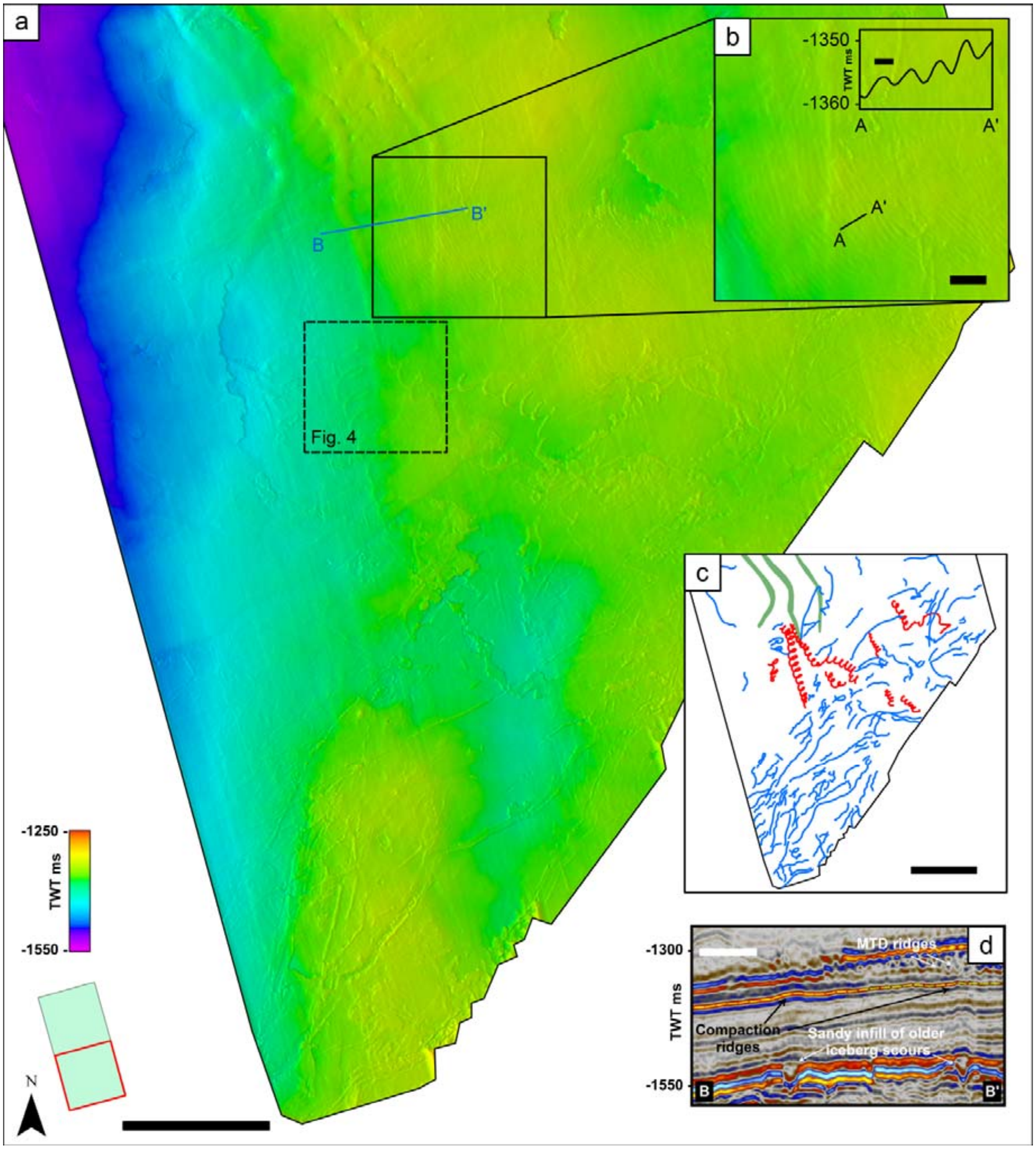

Supplementary Figure 2:

Enlarged structure map. **a.** Shaded relief surface showing TWT depth across the ~430 ka palaeo-seafloor investigated here. Surface is illuminated from the southeast and the colour bar represents TWT values across the 3D surface. Notice the arcuate and westerly

propagating ridges in the central part of the northern area which are related to a mass transport deposit. It is important to note that not all of the iceberg scours are visible at this scale due to the subtlety of their vertical profiles. **b.** arcuate ridge-trough texture due to interference with an overlying mass transport deposit (MTD). **c.** line drawing showing digitised spiral (red) and curvilinear (blue) scours. Also displayed are the compaction ridges (green polygons) that are described in panels (b) and (d). **d.** annotated seismic section showing the impact of differential compaction across sand-filled giant iceberg scours beneath the spiral iceberg scoured surface and also MTD ridges from above. The yellow strippled line is the surface with the spiral iceberg scours. For location see panel (a). Black bar is scale bar in panel (a) of 5 km; panel (b) the bottom right is 1 km and the bar with the elevation profile is 100 m; panel (c) is 5 km; panel (d) is 1 km.

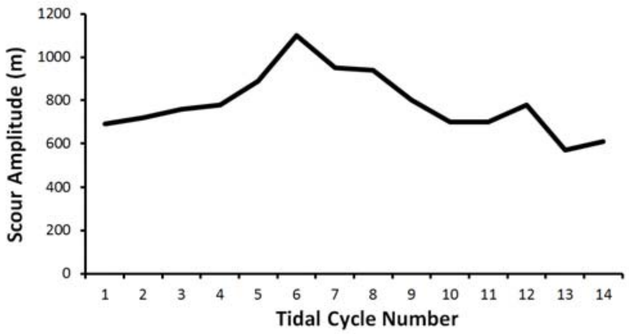

Supplementary Figure 3:

Spring tide iceberg scouring. Graph showing the evolution of tidal amplitude estimated from scour 2. Note the gentle rise to a peak before dropping away again. This is interpreted as a time series of the spring tide. The asymmetry either side of the central peak is possibly related to localised weather or iceberg melt that influences how the iceberg moves through the water and skews amplitude values on separate days.

Scour	Length (km)	Number of tidal cycles	Minimum Wavelength (m)	Maximum Wavelength (m)	Mean Wavelength (m)	Minimum Amplitude (m)	Maximum Amplitude (m)	Mean Amplitude (m)	Minimum Translatory Velocity (cm s^{-1})	Maximum Translatory Velocity (cm s^{-1})	Mean Translatory Velocity (cm s^{-1})	Minimum Tidal Velocity (cm s^{-1})	Maximum Tidal Velocity (cm s^{-1})	Mean Tidal Velocity (cm s^{-1})
**1**	7.5	2	1190	1190	1190	890	890	890	2.7	2.7	2.7	4.0	4.0	4.0
**2**	31.5	15	410	860	669 ± 42	570	1100	785 ± 38	0.9	1.9	1.5 ± 0.1	2.6	4.9	3.5 ± 0.2
**3**	30.9	19	350	1330	653 ± 59	240	1150	592 ± 51	0.8	3.0	1.5 ± 0.1	1.1	5.1	2.6 ± 0.2
**4**	5.6	6	370	540	411 ± 26	200	400	284 ± 32	0.8	1.2	0.9 ± 0.1	0.9	1.8	1.3 ± 0.1
**5**	18.4	4	625	870	763 ± 72	600	740	683 ± 43	1.4	1.9	1.7 ± 0.2	2.7	3.3	3.1 ± 0.2
**6**	7.2	3	640	730	685 ± 45	650	720	685 ± 35	1.4	1.6	1.5 ± 0.1	2.9	3.2	3.1 ± 0.2
**7**	5.5	4	305	560	410 ± 54	400	560	475 ± 34	0.7	1.3	0.9 ± 0.1	1.8	2.5	2.1 ± 0.2
**8**	6.6	5	205	640	419 ± 72	235	520	416 ± 47	0.5	1.4	0.9 ± 0.2	1.1	2.3	1.9 ± 0.2

Supplementary Table 1:

Summary statistics. Statistics from the wavelengths and amplitudes of the tidally-influenced iceberg scours. Error in mean velocity is estimated for the standard error in wavelength and amplitude (see Fig. 5b).
